# Targeted Therapy and Immunotherapy for Melanoma in Japan

**DOI:** 10.1007/s11864-019-0607-8

**Published:** 2019-01-24

**Authors:** Kenjiro Namikawa, Naoya Yamazaki

**Affiliations:** Department of Dermatologic Oncology, National Cancer Center Hospital, 5-1-1 Tsukiji, Chuo-ku, Tokyo, 104-0045 Japan

**Keywords:** Melanoma, Targeted therapy, Immunotherapy, Acral, Mucosal, Asian

## Abstract

Melanoma has several clinically and pathologically distinguishable subtypes, which also differ genetically. Mutation patterns vary among different melanoma subtypes, and efficacy of immune-checkpoint inhibitors differs depending on the subtype of melanoma. In spite of the recent revolution of systemic therapies for advanced melanoma, access to innovative agents is still restricted in many countries. This review article aimed to describe the epidemiology and current status of systemic therapies for melanoma in Japan, where melanoma is rare, but access to innovative agents is available. Acral and mucosal melanomas, which are common in Asian populations, predominantly occur in sun-protected areas and share several biological features. Both the melanomas harbor KIT mutation in approximately 15% of the cases; BRAF or NRAS mutation is found in approximately 10–15% of acral melanoma, but these mutations are less frequent in mucosal melanoma. Combined use of BRAF and MEK inhibitors is one of the standards of care for patients with advanced BRAF-mutant melanoma. In patients with melanoma harboring KIT mutation in exon 11 or 13, KIT inhibitors can be a treatment option; however, none of them have been approved in Japan. Immune-checkpoint inhibitors are expected to be less effective against acral and mucosal melanomas because their somatic mutation burden is lower than those in non-acral cutaneous melanomas. A recently completed phase II trial of nivolumab and ipilimumab combination therapy in 30 Japanese patients with melanoma, including seven with acral and 12 with mucosal melanoma, demonstrated an objective response rate of 43%. Regarding oncolytic viruses, canerpaturev (C-REV, also known as HF10) and talimogene laherparepvec (T-VEC) are currently under review in early phase trials. In the adjuvant setting, dabrafenib plus trametinb combination, nivolumab monotherapy, and pembrolizumab monotherapy were approved in July, August, and December 2018 in Japan, respectively. However, most of the adjuvant phase III trials excluded patients with mucosal melanoma. A phase III trial of adjuvant therapy with locoregional interferon (IFN)-β versus surgery alone is ongoing in Japan (JCOG1309, J-FERON), in which IFN-β is injected directly into the site of the primary tumor postoperatively, so that it would be drained through the untreated lymphatic route to the regional node basin. After the recent approval of these new agents, the JCOG1309 trial will be revised to focus on patients with stage II disease. In conclusion, acral and mucosal melanomas have been treated based on the available medical evidence for the treatment of non-acral cutaneous melanomas. Considering the differences in genetic backgrounds and therapeutic efficacy of immunotherapy, specialized therapeutic strategies for these subtypes of melanoma should be established in the future.

## Introduction

Systemic therapies for advanced melanoma have been dramatically revolutionized by the development of targeted therapies, such as BRAF and MEK inhibitors, and immunotherapies, such as anti-PD-1 antibodies alone or in combination with anti-CTLA-4 antibody. Although these new agents have become the recommended up-front therapies in several international melanoma guidelines, a recently reported web-based worldwide survey revealed that access to these innovative agents is still restricted in many countries [1].

Melanoma has the following clinically distinguishable subtypes: cutaneous, mucosal, uveal, and unknown primary melanomas. Cutaneous melanomas are further categorized into superficial spreading melanoma (SSM), nodular melanoma (NM), lentigo maligna melanoma (LMM), and acral lentiginous melanoma (ALM) [2] based on their clinical and pathological features. Recent advances in molecular biology have revealed that these subtypes are also genetically distinct [3]. Of these subtypes, ALM and mucosal melanoma occur in sun-protected areas and share several biological characteristics.

In Japan, a combination of dabrafenib and trametinib, pembrolizumab monotherapy, and nivolumab alone or in combination with ipilimumab are currently employed for the treatment of melanoma; and there is a high incidence of ALM and mucosal melanoma in the Japanese population [4••]. Therefore, this review article aimed to describe the epidemiology and current status of systemic therapies for melanoma in Japan, where melanoma is rare, but access to innovative agents is available.

### Epidemiology of melanoma in Japan

According to nationwide statistical surveys on skin malignancies in Japan, among the types of skin cancers including both melanomas and non-melanoma skin cancers, basal cell carcinoma (BCC) was the most common, followed by cutaneous squamous cell carcinoma (cSCC), cutaneous melanoma, and extra-mammary Paget’s disease. When carcinoma in situ is included, such as actinic keratosis and Bowen’s disease, cSCC would be the most common [5]. Melanoma is the third most common type of skin cancer, but comprising approximately half of all deaths from skin cancers in Japan; thus, it is considered to be the most common cause of death from skin cancers.

**Fig. 1:**
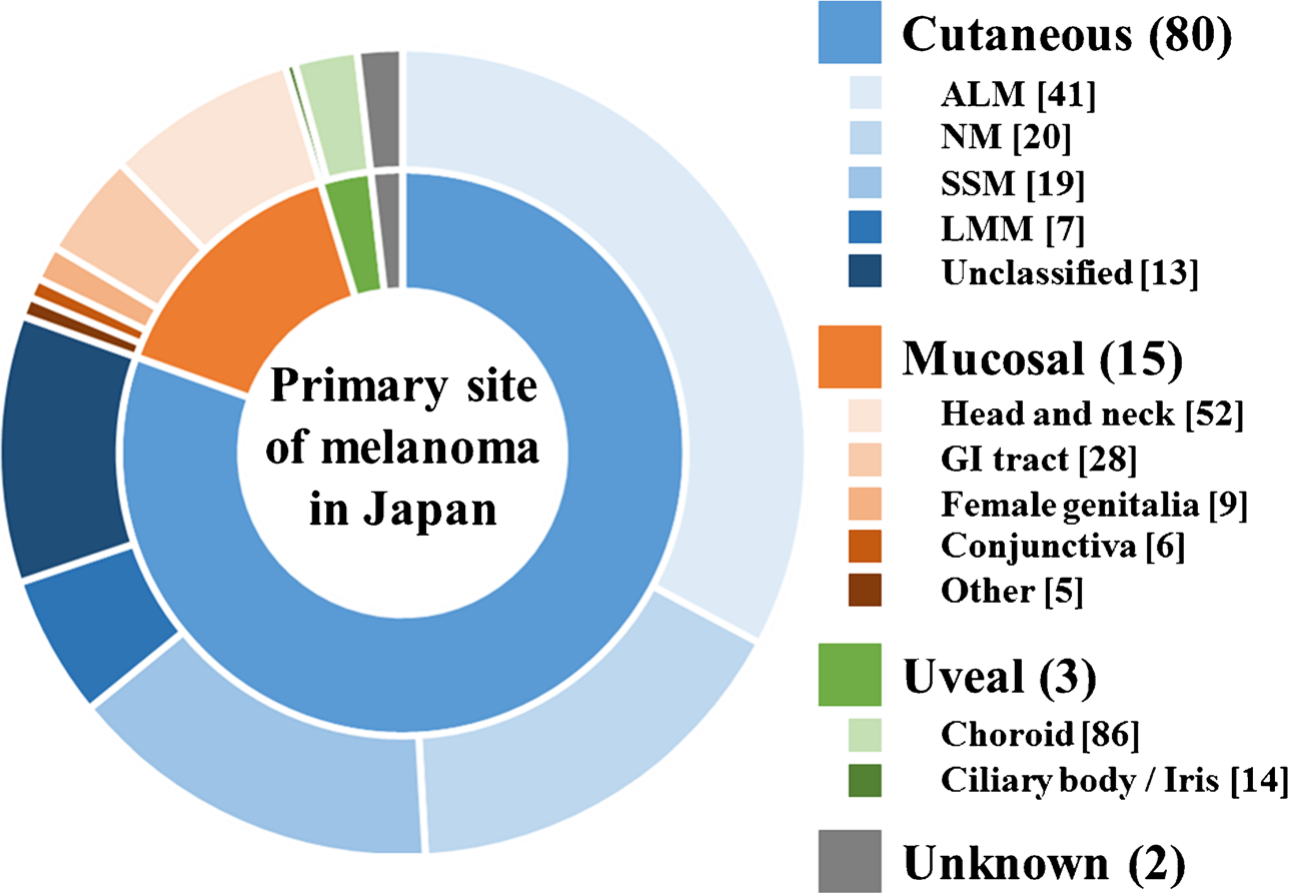
Primary sites and subtypes of melanoma in Japan, created from the statistics based on Hospital-Based Cancer Registries and nationwide statistical surveys [4, 5]. The numbers in round brackets refer to proportion among all types of melanoma. The numbers in square brackets refer to proportion among each subtype of melanoma. Abbreviations: ALM, acral lentiginous melanoma; NM, nodular melanoma; SSM, superficial spreading melanoma; LMM, lentigo maligna melanoma; GI, gastrointestinal

A recent statistics according to Hospital-Based Cancer Registries showed that the proportion relative to all types of melanoma and the crude incidence rate per 100,000 person-years for each subtype were as follows: cutaneous, 80.5% and 1.75; mucosal, 14.8% and 0.32; uveal, 2.9% and 0.064; and unknown primary melanoma, 1.8% and 0.039, respectively [4••]. The greater proportion of mucosal melanoma among all melanomas has been considered to be due to the low incidence of non-acral cutaneous melanoma in Asian populations. However, the study demonstrated that the incidence rate of mucosal melanoma might also be higher in Asian populations. Mucosal melanoma is primarily located in the head and neck regions in 52%, gastrointestinal tract in 28%, female genitalia in 9%, conjunctiva in 6%, and others in 5% of cases [4••]. The ALM subtype of cutaneous melanoma was observed in 41%, NM in 20%, SSM in 19%, LMM in 7%, and unknown or unclassified in 13% of cases [5] (Fig. 1). Only 4% of cutaneous melanomas are stage IV at diagnosis, whereas the proportion of patients with metastatic tumor at diagnosis in mucosal melanoma is as high as 25% [4••]; thus, mucosal melanoma comprised nearly 40% of all metastatic melanomas in Japan.

### Targeted therapies for metastatic melanoma

Researchers with The Cancer Genome Atlas (TCGA) Network identified four genomic subgroups of melanoma: BRAF, NRAS, NF1, and triple wild-type [6]. The mutation patterns vary among the melanoma subtypes, both in terms of the total numbers of mutations and the types of driver oncogenes [7, 8•]. KIT mutations were found in approximately 15% of acral and mucosal melanomas, and BRAF or NRAS mutation was found in approximately 10–15% of acral melanoma, but was less frequent in mucosal melanoma [8•, 9]. Acral and mucosal subtypes of melanoma are predominant in the Japanese population, and the overall detection rates of BRAF, NRAS, and KIT mutations are approximately 30%, 12%, and 13%, respectively [10, 11]. Since subungual melanoma specimens obtained from patients undergoing digital amputation are unlikely to have been included in the aforementioned studies due to the decalcification procedures in the bone, the actual detection rate of BRAF mutations in Japanese patients with melanoma may be lower than that reported in those studies.

In acral and mucosal melanomas, KIT has been considered to be one of the reasonable therapeutic targets [12, 13]. A KIT inhibitor, imatinib, demonstrated its clinical efficacy in phase II studies involving patients with KIT mutations [14–16], although it was not effective in another clinical trials that did not include KIT mutation or amplification in the eligibility criteria [17–19]. In a phase II study of 25 patients, six (24%) responders had a mutation with either KIT exon 11 or 13 [14]. In another phase II study of 43 patients, ten (23%) patients responded to imatinib, of these, nine had KIT gene mutations (exon 11 in six and exon 13 in three patients) and the other one had KIT gene amplification [15]. In a phase II study in which seven (29%) of 24 patients responded to imatinib, seven of 13 patients with KIT mutations responded, whereas none of 11 patients with KIT amplification responded [16]. At present, imatinib is likely most effective in patients with melanoma harboring KIT mutations (exon 11 or 13); however, the response rate is not that high, and a durable response seems not to be expected. In Japan, clinical studies focusing on patients with KIT mutations have not yet been conducted.

The activated BRAF-mutated kinase can be inhibited by BRAF inhibitors, and its downstream MEK activation can be inhibited by MEK inhibitors. Combined use of BRAF and MEK inhibitors can delay the emergence of drug resistance against BRAF inhibitors and can also reduce the risk of occurrence of skin toxicities such as cSCC. In patients with advanced melanomas harboring BRAF V600 mutation, three different combinations of BRAF plus MEK inhibitors have been shown to yield superior clinical outcomes over BRAF inhibitor alone: dabrafenib plus trametinib versus dabrafenib (COMBI-d) [20–22], dabrafenib plus trametinib versus vemurafenib (COMBI-v) [23], vemurafenib plus cobimetinib versus vemurafenib (coBRIM) [24, 25], and encorafenib plus binimetinib versus encorafenib or vemurafenib (COLUMBUS) [26, 27]. In Japan, clinical studies on vemurafenib plus cobimetinib combination therapy have not been conducted, but a phase I/II study of vemurafenib monotherapy has been carried out [28]. A total of 11 patients with advanced melanoma were enrolled, with an overall response rate of 55%, and no patients developed cSCC. Vemurafenib monotherapy was approved in Japan in December 2014. Subsequently, a phase I/II trial of dabrafenib plus trametinib combination therapy was carried out on 12 patients with advanced melanoma [29]. The overall response rate was 83%, and the most common adverse event (AE) was pyrexia, which was observed in 75% of the participants. The efficacy and toxicities observed in this study were almost comparable to those reported in the international phase III studies. Dabrafenib plus trametinib combination therapy in the metastatic setting was approved in March 2016 in Japan. Japanese institutions also participated in the COLUMBUS trial, and the combination of encorafenib plus binimetinib was approved in January 2019 (Table 1). Some clinical trials comparing sequential regimens of BRAF plus MEK inhibitors followed by nivolumab plus ipilimumab combination with the reverse sequence are ongoing. Other clinical trials on concurrent BRAF plus MEK inhibitors with anti-PD-1 or anti-PD ligand 1 (PD-L1) antibodies are also ongoing. Of these, Japanese institutions are currently involved in the COMBI-i trial that evaluates BRAF plus MEK inhibitors along with the concurrent use of anti-PD-1 antibody (ClinicalTrials.gov Identifier: NCT02967692).

**Table 1:** Pivotal international phase III studies and corresponding Japanese studies on targeted therapy and immunotherapy for melanoma

Agents	Pivotal P3 studies	Corresponding Japanese studies
**Metastatic setting**		
Molecularly targeted agents		
Vemurafenib	BRIM-3 [30, 31]	JO28178 (P1/2) [32]
Dabrafenib	BREAK-3 [33]	BRF116056 (P1) [34]
Trametinib	METRIC [35]	MEK114784 (P1) [36]
Vemurafenib + Cobimetinib	coBRIM [24, 25]	Not done
Dabrafenib + Trametinib	COMBI-v [23], -d [20–22]	MEK116885 (P1/2) [29]
Encorafenib + Binimetinib	COLUMBUS [26, 27]	
Immune checkpoint inhibitors		
Ipilimumab (3 mg)	CA184-002 [37]	CA184-396 (P2) [38]
Ipilimumab (10 mg)	CA184-024 [39]	CA184-202 (P2) [40]
Nivolumab (second line)	CheckMate 037 [41]	ONO-4538-02 (P2, 2 mg Q3w) [42]
Nivolumab (first line)	CheckMate 066 [43]	ONO-4538-08 (P2, 3 mg Q2w) [44]
Pembrolizumab	KEYNOTE-006 [45, 46]	KEYNOTE-041 (P1b) [47]
Nivolumab + Ipilimumab	CheckMate 067 [48, 49]	ONO-4538-17 (P2) [50•]
**Adjuvant setting**		
Molecularly targeted agents		
Vemurafenib	BRIM-8 [51]	Not done
Dabrafenib + Trametinib	COMBI-AD [52, 53]	
Immune checkpoint inhibitors		
Ipilimumab (10 mg)	EORTC 18071 [54, 55]	Not done
Ipilimumab (3/10 mg)	E1609 [56]	Not done
Nivolumab	CheckMate 238 [57]	
Pembrolizumab	EORTC 1325/KEYNOTE-054 [58]	
Interferons		
Pegylated interferon alfa	EORTC 18991 [59, 60]	MK-4031-370 (P1) [61]
Interferon beta	Not done	JCOG1309/J-FERON (P3) [62•]

P, phase; Q2w, every 2 weeks; Q3w, every 3 weeks

NRAS mutation is found in approximately 15% of acral melanoma, but it is rarely seen in mucosal melanoma [8•]. In patients with NRAS-mutant melanoma, a phase III randomized controlled trial assigned either MEK inhibitor, binimetinib, or dacarbazine (DTIC) at a ratio of 2:1 (NEMO). Japanese institutions also participated in the NEMO trial. The results revealed a significantly better median progression-free survival (PFS) in the binimetinib group (2.8 vs. 1.5 months; hazard ratio [HR], 0.62; 95% confidence interval [CI], 0.47–0.80; *p* < 0.001), but no statistically significant difference was noted in the median overall survival (OS) between the groups (11.0 vs. 10.1 months; HR, 1.00; 95% CI, 0.75–1.33; *p* = 0.499) [63]. Other clinical trials of combination therapy, such as MEK inhibitor plus cyclin-dependent kinase 4/6 inhibitor or MEK inhibitor plus anti-PD-1/PD-L1 antibody, are ongoing; however, no Japanese institutions are involved.

### Immunotherapies for metastatic melanoma

The efficacy of immune-checkpoint inhibitors, such as ipilimumab (anti-CTLA-4 antibody) and nivolumab or pembrolizumab (anti-PD-1 antibodies), have been demonstrated to be superior to that of the conventional agents. Pembrolizumab was associated with a significantly prolonged PFS and OS as compared to ipilimumab in the KEYNOTE-006 randomized phase III trial [45, 46], and nivolumab followed by ipilimumab therapy was associated with improved outcomes as compared to treatment in the reverse sequence in the CheckMate 064 randomized phase II trial [64]. The clinical outcomes of nivolumab alone or in combination with ipilimumab were shown to be superior to that of ipilimumab monotherapy in the CheckMate 067 randomized phase III trial [48, 49]. Therefore, nivolumab monotherapy, pembrolizumab monotherapy, and nivolumab combined with ipilimumab are the currently available first choices of systemic immunotherapy in patients with advanced melanoma.

The efficacy of immune-checkpoint inhibitor differs depending on the subtype of melanoma; for example, the efficacy of immune-checkpoint inhibitor monotherapy on metastatic uveal melanoma has been shown to be limited [65–71]. Melanomas occurring in sun-exposed skin tend to have higher numbers of mutations, whereas those occurring in sun-protected areas, such as mucosal or acral melanomas, have significantly lower total numbers of single-nucleotide variants [72••]. Immune-checkpoint inhibitors are known to be more effective against tumors with a higher mutation burden; therefore, they are expected to be less effective against acral and mucosal melanomas. A multi-institutional retrospective study evaluated the clinical efficacy of anti-PD-1 antibody in 25 and 35 patients with acral and mucosal melanoma, respectively. In this study, 51 patients (85%) had a previous history of systemic therapies, including ipilimumab. The objective response rate and median PFS were 32% and 4.1 months in patients with acral melanoma, and 23% and 3.9 months in those with mucosal melanoma, respectively [73]. Another study evaluated the efficacy and safety of nivolumab alone or in combination with ipilimumab for mucosal melanoma, using data pooled from several clinical trials of nivolumab monotherapy or nivolumab plus ipilimumab combination therapy. The objective response rate and median PFS in nivolumab monotherapy in patients with mucosal melanoma were 23% and 3.0 months and those in the nivolumab and ipilimumab combination therapy were 37% and 5.9 months, respectively [74]. In a post hoc analysis of clinical trials on pembrolizumab monotherapy, the objective response rate, median PFS, and median OS associated with pembrolizumab monotherapy in 84 patients with mucosal melanoma were 19%, 2.8 months, and 11.3 months, respectively [75]. These studies consistently demonstrated a certain efficacy of immune-checkpoint inhibitors against acral or mucosal melanoma; however, these therapies appear to be somewhat less efficacious than those against non-acral cutaneous melanomas.

In Japan, a phase II trial of ipilimumab 10 mg/kg plus dacarbazine was terminated early due to the high frequency of liver toxicity [40]. A subsequent phase II trial of ipilimumab 3 mg/kg monotherapy was completed, which showed an acceptable tolerability. The proportion of patients who developed immune-related AE in this trial was 60%. The best overall response and disease control rates were 10% and 20%, respectively [38]. Phase II trials of nivolumab (2 mg/kg, every 3 weeks) in patients with past history of systemic therapies [42] and nivolumab (3 mg/kg, every 2 weeks) in previously untreated patients [44] were conducted; in the former trial, in the second-line setting, 35 evaluable patients were enrolled. The best overall response rate was 29%, median PFS was 5.6 months, and median OS was 18.0 months. Grades 3–4 drug-related AEs were observed in 31% of patients [42]. In the latter trial, in the first-line setting, 24 patients were enrolled. The best overall response rate was 35%, median PFS was 5.9 months, and median OS was not reached. Grades 3–4 drug-related AEs were observed in 13% of patients [44]. A phase Ib trial of pembrolizumab was also carried out on 42 patients with 0–2 prior lines of systemic therapy, including 12 patients with ALM and eight patients with mucosal melanoma [47]. Among the 37 evaluable patients, the confirmed overall response rate was 24% in those with cutaneous melanoma and 25% in those with mucosal melanoma. A phase II trial of nivolumab and ipilimumab combination therapy was recently completed [50•]. A total of 30 patients, including seven with acral and 12 with mucosal melanoma, were enrolled. The overall response rate was 43%, and neither the median PFS nor OS was reached. Grades 3–4 and serious AEs occurred in 23 (77%) and 20 (67%) patients, respectively. This study confirmed a certain efficacy and safety of nivolumab plus ipilimumab in previously untreated Japanese patients with advanced melanoma, including rare subtypes (Table 1). Although the clinical efficacy of immune-checkpoint inhibitors has been shown to be somewhat lower in Japanese patients with melanoma than those in Caucasians, whether the differences are due to melanoma subtypes alone or also due to the ethnicity remains unclear.

Oncolytic viruses are injected directly into the tumor to promote the release of tumor-associated antigens and mediate the host’s antitumor immunity. Talimogene laherparepvec (T-VEC) is a genetically modified herpes simplex virus-1, and T-VEC monotherapy demonstrated a durable response rate of 16% in the OPTiM phase III trial [76]. In that trial, responses are also observed in uninjected lesions including visceral sites [77]. In Japan, a multicenter phase I trial of T-VEC for melanoma is ongoing (NCT03064763). Another oncolytic virus, canerpaturev (C-REV, also known as HF10), is an attenuated, replication-competent mutant strain of herpes simplex virus-1. In Japan, a single-institutional phase I study of C-REV monotherapy was completed (NCT02428036), and a subsequent multicenter phase II study of C-REV combined with ipilimumab has also been completed (NCT03153085).

### Adjuvant therapies for melanoma

Results of several clinical trials on new agents, such as immune-checkpoint inhibitors and BRAF/MEK inhibitors, in the adjuvant setting have been published. The EORTC18071 trial, a randomized phase III trial of adjuvant therapy with ipilimumab administered at the dose of 10 mg/kg in patients with postoperative stage III cutaneous melanoma, showed a significantly longer OS in the ipilimumab arm than in the placebo arm [54, 55]. However, adjuvant ipilimumab administered at 10 mg/kg was associated with high frequency of toxicities, including five (1.1%) treatment-related deaths. The COMBI-AD trial, in which patients with stage III disease (stage IIIA melanoma with a tumor deposit of > 1 mm diameter within the sentinel lymph node (SLN)) were assigned to either dabrafenib plus trametinib or placebo, showed a significantly longer relapse-free survival (RFS) in the dabrafenib plus trametinib treatment arm (HR 0.47; 95% CI, 0.39 to 0.58; *p* < 0.001) [52, 53]. The CheckMate238 trial, in which patients with stage III disease (stage IIIA melanoma with a tumor deposit of > 1 mm in diameter within the SLN) were assigned to either nivolumab 3 mg/kg or ipilimumab 10 mg/kg, showed a significantly longer RFS and more favorable toxicity profile in the nivolumab treatment arm (HR 0.65; 97.56% CI, 0.51 to 0.83; *p* < 0.001) [57]. Subsequently, the EORTC1325/KEYNOTE-054 trial, in which patients with stage III disease (stage IIIA melanoma with a tumor deposit of > 1 mm diameter within the SLN) were assigned to either pembrolizumab 200 mg/body or placebo, showed a significantly longer RFS in the pembrolizumab treatment arm (HR 0.57; 98.4% CI, 0.43 to 0.74; *p* < 0.001) [58]. Japanese institutions have participated in the COMBI-AD, CheckMate238, and EORTC1325/KEYNOTE-054 trials. In Japan, dabrafenib plus trametinib combination, nivolumab monotherapy, and pembrolizumab monotherapy were approved as adjuvant therapy in July, August, and December 2018, respectively.

Among these recent trials in the adjuvant setting, patients with mucosal melanoma were included only in the CheckMate238 trial, although the number of patients included was limited. In patients with mucosal melanoma, results of a phase II trial of adjuvant therapy have been published from China [78]. Patients with resected mucosal melanoma were randomized to observation, high-dose interferon (IFN)-alfa, or chemotherapy with temozolomide plus cisplatin. Patients in the chemotherapy arm showed a significantly longer RFS and OS than those in either the high-dose IFN-alfa or observation arm. Results of a subsequently conducted phase III trial comparing high-dose IFN-alfa with temozolomide plus cisplatin were presented at the American Society of Clinical Oncology (ASCO) 2018; they confirmed the superiority, in terms of the RFS, of temozolomide plus cisplatin over high-dose IFN-alfa [79]. However, no formal confirmatory trials of immune-checkpoint inhibitors for mucosal melanoma have been reported yet.

Before the recent breakthroughs in systemic therapies for melanoma, IFNs were the most frequently used agents in the adjuvant setting. High-dose or pegylated IFN-alfa has been shown to improve the RFS; however, its efficacy in terms of the OS is marginal, and the incidence of severe AEs is relatively high [59, 60, 80–82]. In Japan, pegylated-IFN-alfa was approved in May 2015 after the completion of a phase I trial [61]. Another type I IFN, IFN-β, has also been used for melanoma in Japan. The lymphatic route between the primary site and the regional node basin usually remains untreated even after a definitive curative surgery because the primary site of cutaneous melanoma is often located away from its regional node basin. To improve the outcome of adjuvant IFN therapy and lower its toxicity, the Dermatologic Oncology Group of Japan Clinical Oncology Group (JCOG-DOG) began a phase III randomized controlled trial of adjuvant therapy with locoregional IFN-β versus surgery alone in patients with stage II/III cutaneous melanoma (JCOG1309, J-FERON trial) in May 2015 [62•]. In this trial, IFN-β was injected directly into the surgical site of the primary tumor postoperatively, similar to the procedure used for SLN biopsy, so that it would be drained through the untreated lymphatic route to the regional node basin. The JCOG-DOG hypothesized that the locally injected IFN-β would reach clinically occult residual melanoma cells along the untreated lymphatic route and possibly induce systemic immunity. After the recent approval of new agents in the adjuvant setting in Japan, such as dabrafenib plus trametinib combination, nivolumab monotherapy, or pembrolizumab monotherapy, the JCOG-DOG plans to revise the protocol to focus on patients with stage II disease.

## Conclusions

Acral and mucosal melanomas have been treated based on the available medical evidence for the treatment of non-acral cutaneous melanomas. Considering the differences in genetic backgrounds and therapeutic efficacy of immunotherapy, specialized therapeutic strategies for these subtypes of melanoma should be established in the future.
